# Harvard Odontological Society

**Published:** 1897-08

**Authors:** 

**Affiliations:** Harvard Odontological Society


### HARVARD ODONTOLOGICAL SOCIETY.

A REGULAR meeting of the Society was held on Thursday evening, December 3, 1896, at Young's Hotel, the President, Dr. Waldo E. Boardman, in the chair.

The President called upon Dr. George F. Grant to give his "Description of some English Appliances."

(For Dr. Grant's paper, see page 497.)

*Dr. Grant.*—The appliances that I am going to present to you to-night are the outcome of a visit from a friend of mine about a year ago, a dentist who was taking a little vacation from his practice in England. We sat down and compared notes, and each told of things which he thought might interest the other, and some of the things that he mentioned he said he would send over that I might try them.

The first one is a little matrix, called the "Lennox matrix." It is a very ingenious little thing and is quite simple. I cannot say that I have handled all the matrices that have been in use, but I can say that this is the best and easiest to handle of any that I have yet tried. Take a straight band matrix and it never fits a tooth, but this one, being cut on the segment of a circle, fits almost anything. I will pass one of the bands around, bent in position to be applied. All of these have a little mark, a black line, that corresponds to the form of the circle, so that when the band enters the lug there the strain is in a line with the form of the tooth, and by simply turning up the screw it fits at once. The matrix is so thin that it will pass between almost any teeth.

The next thing is some of the uses of fusible metal. Everybody who has had artificial work to do appreciates the difficulty of getting a base-plate which has some approximation to the finished plate. I do not know of anything which can be used with perfect satisfaction as a base-plate that can be prepared quickly, whereupon he suggested and sent to me what is called "King's modelling outfit." The process is very much like ours, except that fusible metal is used directly from the modelling composition. Of course, you have your model, which may be simply a wax pattern, but with a little knob on the back of it the same as if you were preparing the finished plate. The metal is placed in a ladle and set in boiling water. It fuses at the point of boiling water, and you pour it right in there, and in a minute it hardens so that you can remove it. That will tell you exactly within a short time whether there is sufficient atmospheric pressure on the back of the plate, and it is very often quite important to determine this at once when you intend making a set of teeth. You get an accurate bite, and have a plate to work on that is rigid, not very thick, and offers good resistance.

*Dr. Smith.*—What do you grind your teeth to,—the paraffin or the fusible metal?

*Dr. Grant.*—Sometimes you can finish the pattern right down and use it as a base-plate.

*Dr. Smith.*—Do away with the model and continue with the case with the fusible metal plate?

*Dr. Grant.*—Sometimes; not always. It is very often easier in fitting teeth to bury the teeth in wax. The point is the advantage of getting a good-fitting plate at once.

*Dr. Cook.*—You can make it set while you wait.

*Dr. Stoddard.*—How long will such a plate last?

*Dr. Grant.*—Well, that depends. In some cases, where it is handled carefully, it may last very well. In other cases they might need a new one shortly after they paid for it.

Another place where I found this fusible metal quite convenient was in the setting of a crown, where it is desirable to have a sharp cast of the end of the root. I take the impression with modelling compound and pour the metal right into it, and have got a perfect cast that I can swage up on at once. It is easier than changing from a plaster cast to a die, and you have little difficulty in getting a good cast.

It may also be used in preparing a regulating plate where you want it to fit quickly. It is a great deal better sometimes to get up a die, where the teeth are crooked, for caps on the teeth. You can do that at once.

*Dr. Stoddard.*—What did you call it?

*Dr. Grant.*—Lennox fusible metal. You can get it at Ash's. One formula is five parts bismuth, three parts lead, two parts tin. It fuses at the point of boiling water. You can get it a little lower with antimony, but it is not necessary.

*Dr. Parsons.*—How many times can you use that metal?

*Dr. Grant.*—Oh, indefinitely.

*Dr. Stoddard.*—Do you always melt it in boiling water rather than over a direct flame?

*Dr. Grant.*—Yes, because the bismuth will burn out easily if you get it over the melting-point.

*Dr. Stoddard.*—How do you separate it from the model?

*Dr. Grant.*—It comes right off. The surface of the modelling composition will soften in the heat of the metal, and you can take it off in an instant.

*Dr. Stoddard.*—What did you say was the name of the modelling composition?

*Dr. Grant.*—King's compound. It takes a sharper impression of the model than anything I have seen. It is used in the same way as the ordinary red modelling composition.

The other thing that I have brought down I do not think, perhaps, many of you will care about; but in making a metallic plate it is quite desirable very often to have a thin cast, and you can get a thinner cast, and one that you can remove readily from sand. This modelling flask of Dr. Pearsall I find very convenient. It has a round, cone-shaped base for the die that insures striking in the centre. Every one who has done any swaging knows that the greatest difficulty is in getting the blow exactly in the centre,—it is very apt to strike one side or the other. I like this flask better than the Hayes. It admits of working very rapidly and yet produces such good results.

I have found all of those things very useful indeed in my practice, and I felt grateful to Dr. Pearsall for showing them to me. He told me that he felt very much gratified with what he saw on this side, but he felt that he would like the Yankees to see that they had good ideas over there.

*Dr. Smith.*—I will speak a few minutes on this modelling flask. When I took the chair of mechanical dentistry at the school, I went to work to brush up on mechanical dentistry, and of course came in touch with this Pearsall flask, and it seemed to me the best thing that there was in the market. It was simple, and yet it seemed, like many English appliances, a little clumsy, and I think that Dr. Grant will agree with me that it takes a large amount of sand. I felt that there might be something better secured for the purposes for which it is used, so I studied on it myself, and also got the wizard of my office, Dr. Shaw, interested in the subject, and with his practical ability he developed a flask that I think is far ahead of anything in the market. I exhibited it to the class last year for the first time, and we are having the patterns made now from which a number can be produced for those who would like them. Had I known that Dr. Grant was to refer to this subject, I would have brought the flask here and shown it. I hardly think that I could describe it to you, but will bring it here at some future meeting.

*Dr. Grant.*—I can quite understand how Dr. Smith, or any one else, could have improved upon that flask in that way, but inasmuch as I received it in the way I did, I did not wish to make any criticism upon it. In fact, the only criticism that I should make would be that it is a little larger than it need be, and yet, under certain circumstances, I find a large body of sand quite desirable. Of course, the Hayes flask is much smaller, and I thought this one better than the Hayes. It served me very well.

He gave me some very good hints in the application of fusible metal. I found it of immense advantage in working where time is an object. They also use it for making splints. They make a splint for a fracture in about an hour, whereas if you have to vulcanize one, it will take the best part of a day. You can see that the great rapidity with which it can be worked is an immense advantage.

*Dr. Smith.*—Can Dr. Grant tell us if, in the use of fusible metal for fractured jaws, the metal is sufficiently strong for these arm-attachments that it is necessary to bring out around the jaws as a support?

*Dr. Grant.*—Yes. It is brittle and will not bend, and yet it is hard enough so that arms of wire are securely retained.

H. L. Upham, D.M.D.,
*Editor Harvard Odontological Society.*

